# A systematic two-sample and bidirectional MR process highlights a unidirectional genetic causal effect of allergic diseases on COVID-19 infection/severity

**DOI:** 10.1186/s12967-024-04887-4

**Published:** 2024-01-23

**Authors:** Xiao-tong Chen, Shuai Zhi, Xin-yu Han, Jian-wei Jiang, Guang-ming Liu, Shi-tao Rao

**Affiliations:** 1 Department of Bioinformatics, Fujian Key Laboratory of Medical Bioinformatics, Institute of Precision Medicine, School of Medical Technology and Engineering, Fujian Medical University, No. 1 Xue-Yuan Rd., University Town, Fuzhou, 350122 Fujian China; 2 Xiamen Key Laboratory of Marine Functional Food, College of Ocean Food and Biological Engineering, Fujian Provincial Engineering Technology Research Center of Marine Functional Food, Jimei University, Xiamen, 361021 Fujian China; 3 School of Biomedical Sciences, The Chinese University of Hong Kong, Shatin, New Territories, Hong Kong

**Keywords:** Allergic diseases, Asthma, COVID-19 infection/severity, GWAS summary statistics, Mendelian randomization, Molecular mechanism, Immune-related cells, Hematological traits

## Abstract

**Background:**

Allergic diseases (ADs) such as asthma are presumed risk factors for COVID-19 infection. However, recent observational studies suggest that the assumed correlation contradicts each other. We therefore systematically investigated the genetic causal correlations between various ADs and COVID-19 infection/severity.

**Methods:**

We performed a two-sample, bidirectional Mendelian randomization (MR) study for five types of ADs and the latest round of COVID-19 GWAS meta-analysis datasets (critically ill, hospitalized, and infection cases). We also further validated the significant causal correlations and elucidated the potential underlying molecular mechanisms.

**Results:**

With the most suitable MR method, asthma consistently demonstrated causal protective effects on critically ill and hospitalized COVID-19 cases (OR < 0.93, *p* < 2.01 × 10^–2^), which were further confirmed by another validated GWAS dataset (OR < 0.92, *p* < 4.22 × 10^–3^). In addition, our MR analyses also observed significant causal correlations of food allergies such as shrimp allergy with the risk of COVID-19 infection/severity. However, we did not find any significant causal effect of COVID-19 phenotypes on the risk of ADs. Regarding the underlying molecular mechanisms, not only multiple immune-related cells such as CD4^+^ T, CD8^+^ T and the ratio of CD4^+^/CD8^+^ T cells showed significant causal effects on COVID-19 phenotypes and various ADs, the hematology traits including monocytes were also significantly correlated with them. Conversely, various ADs such as asthma and shrimp allergy may be causally correlated with COVID-19 infection/severity by affecting multiple hematological traits and immune-related cells.

**Conclusions:**

Our systematic and bidirectional MR analyses suggest a unidirectional causal effect of various ADs, particularly of asthma on COVID-19 infection/severity, but the reverse is not true. The potential underlying molecular mechanisms of the causal effects call for more attention to clinical monitoring of hematological cells/traits and may be beneficial in developing effective therapeutic strategies for allergic patients following infection with COVID-19.

**Graphical Abstract:**

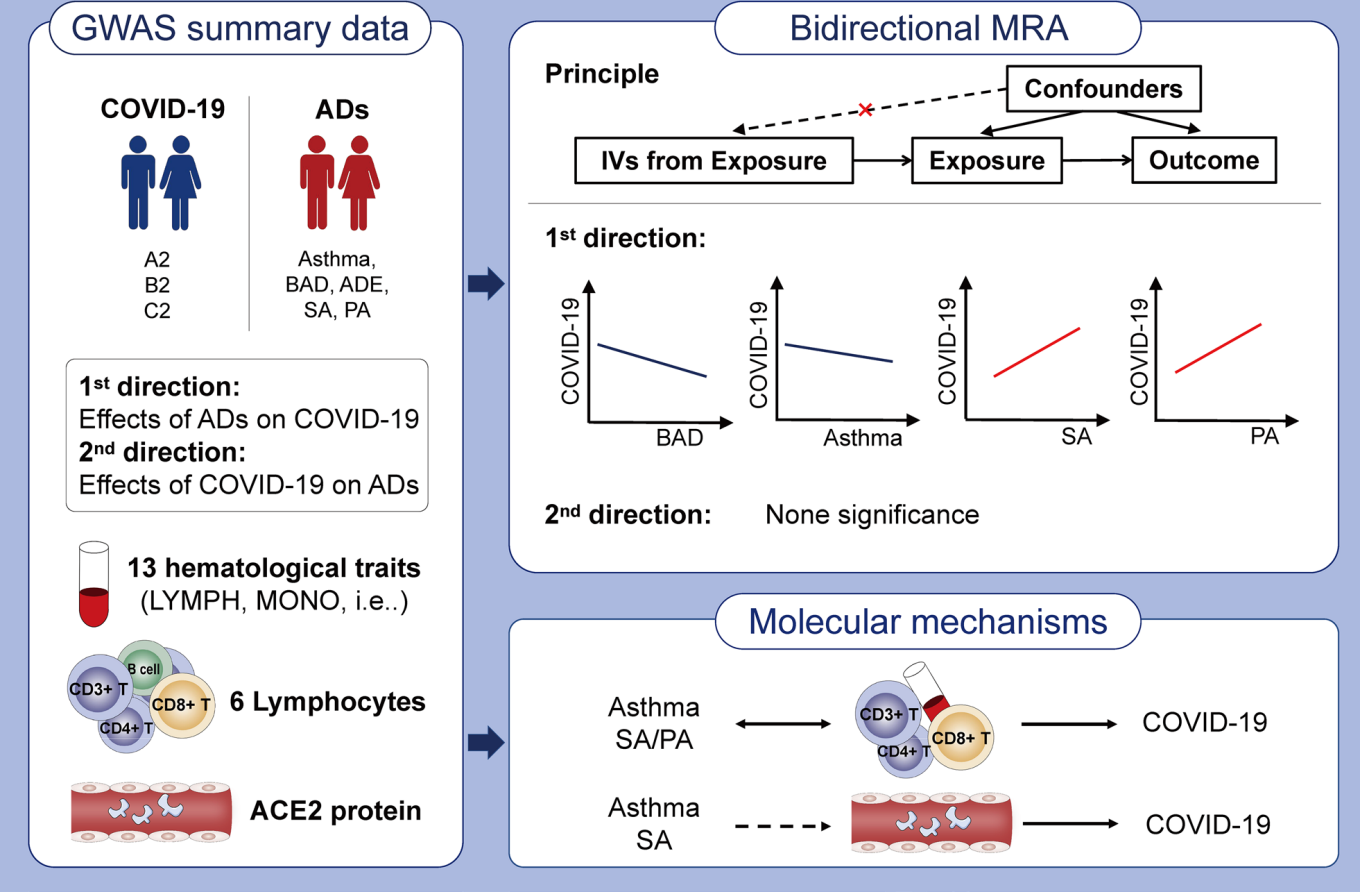

**Supplementary Information:**

The online version contains supplementary material available at 10.1186/s12967-024-04887-4.

## Key messages

1. We performed a two-sample, bidirectional Mendelian randomization study for five types of allergic diseases with COVID-19 infection/severity (critically ill, hospitalized, and infection cases), and also further elucidated the potential underlying molecular mechanisms.
2. Our MR analyses consistently suggest a unidirectional protective causal effect of asthma on COVID-19 infection/severity, but the reverse is not true.
3. Food allergies such as shrimp allergy showed significant causal correlations with the risk of COVID-19 phenotypes.
4. Allergic diseases may be causally correlated with COVID-19 infection/severity by inducing abnormal fluctuations of multiple immune-related cells and hematological traits in peripheral blood.
5. The findings call for more attention to clinical monitoring of hematological traits and may be beneficial in developing effective therapeutic strategies for allergic patients following infection with COVID-19.

**Supplementary Information:**

The online version contains supplementary material available at 10.1186/s12967-024-04887-4.

## Introduction

According to the Coronavirus Disease 2019 (COVID-19) Weekly Epidemiological Update (accessed on 15th May 2023) published by the World Health Organization, approximately 766 million confirmed cases and 6.9 million deaths have been reported globally. It is worth noting that more than one-quarter of COVID-19 cases suffered from at least one comorbid condition (such as diabetes, hypertension, cardiovascular disease, and respiratory diseases) [1, 2] and the disease was more likely to be severe in patients with chronic conditions [3]. Among these comorbid conditions, respiratory diseases attracted the most attention as they showed the most obvious phenotypes associated with COVID-19. Asthma is a common paroxysmal allergic condition partially induced by infection with respiratory viruses [4]. Atopic dermatitis/rhinitis and food allergies can also be caused by an abnormal adaptive immune response to a specific allergen [5, 6].

It is assumed that a close correlation exists between COVID-19 infection and allergic diseases (ADs) as both conditions result from a dysfunctional immune response [7]. However, findings from observational studies about the correlation between COVID-19 infection/severity and ADs contradict each other [6, 8–10] and are under active debate [11–14]. The controversy surrounding traditional observational studies may derive from multiple confounding factors, such as hypertension, diabetes, obesity and other comorbidities in COVID-19 patients [1, 2]. Accumulating evidence have implied that the predisposition to COVID-19 infection and its severity is closely associated with a patient’s age, body mass index (BMI), and smoking status [15–17]. Moreover, results of conventional observational studies are usually affected by reverse causality and, because of that, may not easily be validated via experimental approaches [18]. Although randomized controlled trials (RCTs) are the gold standard for inferring causal relationships, they have been difficult to implement due to high costs and required enrollment of large number of eligible subjects [19].

In recent years, Mendelian randomization (MR) has been used to investigate genetic variants in terms of single-nucleotide polymorphisms (SNPs) or copy number variations as instrumental variables to infer causal correlations between exposures and outcomes [20]. As genetic variants are determined by nature and usually cannot be altered by acquired environmental and behavioral factors, they are considered reliable and practical instrumental variables for inferring causal correlations, which may not be easily affected by issues inherent in observational studies and RCTs [21]. In addition, host genetics has been reported to play a vital role in the predisposition to infectious diseases [22]. Based on the MR approach, severe COVID-19 symptoms were reported to be causally linked to multiple risk factors, including BMI and smoking intensity [23–25]. Our group performed a phenome-wide MR study, which suggested that diabetes and related traits may increase pulmonary angiotensin-converting enzyme 2 (*ACE2*) gene expression, which may in turn increase the rate or severity of COVID-19 infection [26]. However, the potential causal correlation between ADs and COVID-19 infection/severity has been less studied.

In the present study, we aimed to answer the following two questions: (1) Are there causal correlations between various ADs and COVID-19 infection/severity? (2) If so, how do they affect each other? Here, we employed a process of two-sample, bidirectional MR analysis to systematically explore the potential genetic correlations between ADs and COVID-19 infection/severity. Upon obtaining significant correlations, we would further validate the results using another available genome-wide association study (GWAS) summary datasets. We also applied the process to investigate the molecular mechanism underlying the identified significant correlations according to the putative theory about the potential connections between ADs and COVID-19 phenotypes.

## Methods

### GWAS summary statistics

We collated all GWAS summary statistics for COVID-19, ADs, peripheral blood hematological traits, immune-related cell count data, as well as peripheral blood ACE2 protein expression from publicly available databases (Additional file 1: Table S1). Notably, all the GWAS summary data were based on predominantly European samples (> 88%), except for the two GWAS summary datasets of ADs caused by shrimp and peach. Moreover, all the summary data were originally subjected to proper correction regarding population stratification to avoid the negative effects of different genetic backgrounds.

### COVID-19 phenotypes and ADs

We obtained the latest 7th round of COVID-19 Host Genome Initiative GWAS meta-analysis datasets (https://www.covid19hg.org/results/r7/) (Additional file 1: Table S1), which included 18,152 critically ill COVID-19 positive cases and 1,145,546 controls (A2), 44,986 hospitalized COVID-19 positive cases and 2,356,386 controls (B2), and 159,840 reported COVID-19 infection cases and 2,782,977 controls (C2). Critically ill COVID-19 positive cases were defined as laboratory-confirmed SARS-CoV-2 infections AND hospitalization for COVID-19 AND death or respiratory support, while hospitalized COVID-19 positive cases were defined as laboratory-confirmed SARS-CoV-2 infections AND hospitalization for COVID-19. The mildest phenotype (called the reported COVID-19 infection) was defined as laboratory-confirmed SARS-CoV-2 infection OR a COVID-19 diagnosis based on electronic health record/International Classification of Disease coding/physician confirmation OR self-reported COVID-19 via questionnaire. A control was defined as any other person apart from the patients.

Regarding ADs, we comprehensively searched all available databases to systematically investigate correlations between all available types of ADs and COVID-19. Eventually, we obtained seven sets of summary datasets for five types of ADs, including one board allergic disease (BAD, a combination of hay fever, asthma, and eczema), asthma, allergic dermatitis (ADE), shrimp allergy (SA) and peach allergy (PA) (Additional file 1: Table S1) [27–30]. Two of these summary datasets for asthma (Asthma2018) and ADE (ADE2021) were used to validate the primarily significant correlations [31, 32].

### Peripheral blood hematological traits and count data of immune-related cells

We collected a set of 13 different peripheral blood hematological traits, including total blood hemoglobin (HB), red blood cell count, mean corpuscular volume (MCV), platelet count (PLT), white blood cell count, hematocrit (HT), mean cell hemoglobin (MCH), mean corpuscular hemoglobin concentration, and neutrophil (NEUT), monocyte (MONO), eosinophil (EOS), basophil (BASO), and lymphocyte (LYMPH) counts [33] (Additional file 1: Table S1). We also obtained six GWAS summary datasets of count data of immune-related cells, comprising CD3^+^ T cells, CD4^+^ T cells, CD8^+^ T cells, CD19^+^ B cells, CD56^+^ natural killer cells, and the ratio of CD4^+^:CD8^+^ T cells (Additional file 1: Table S1) [34].

### Peripheral blood ACE2 protein expression

Two sets of GWAS summary statistics of soluble ACE2 protein expression level in peripheral blood were obtained for this study. One set was obtained from the KORA (Cooperative Health Research in the Augsburg Region) study [35], and the other from a systematic meta-analysis of 14 European cohorts [36] (Additional file 1: Table S1).

### Study design

**Fig. 1:**
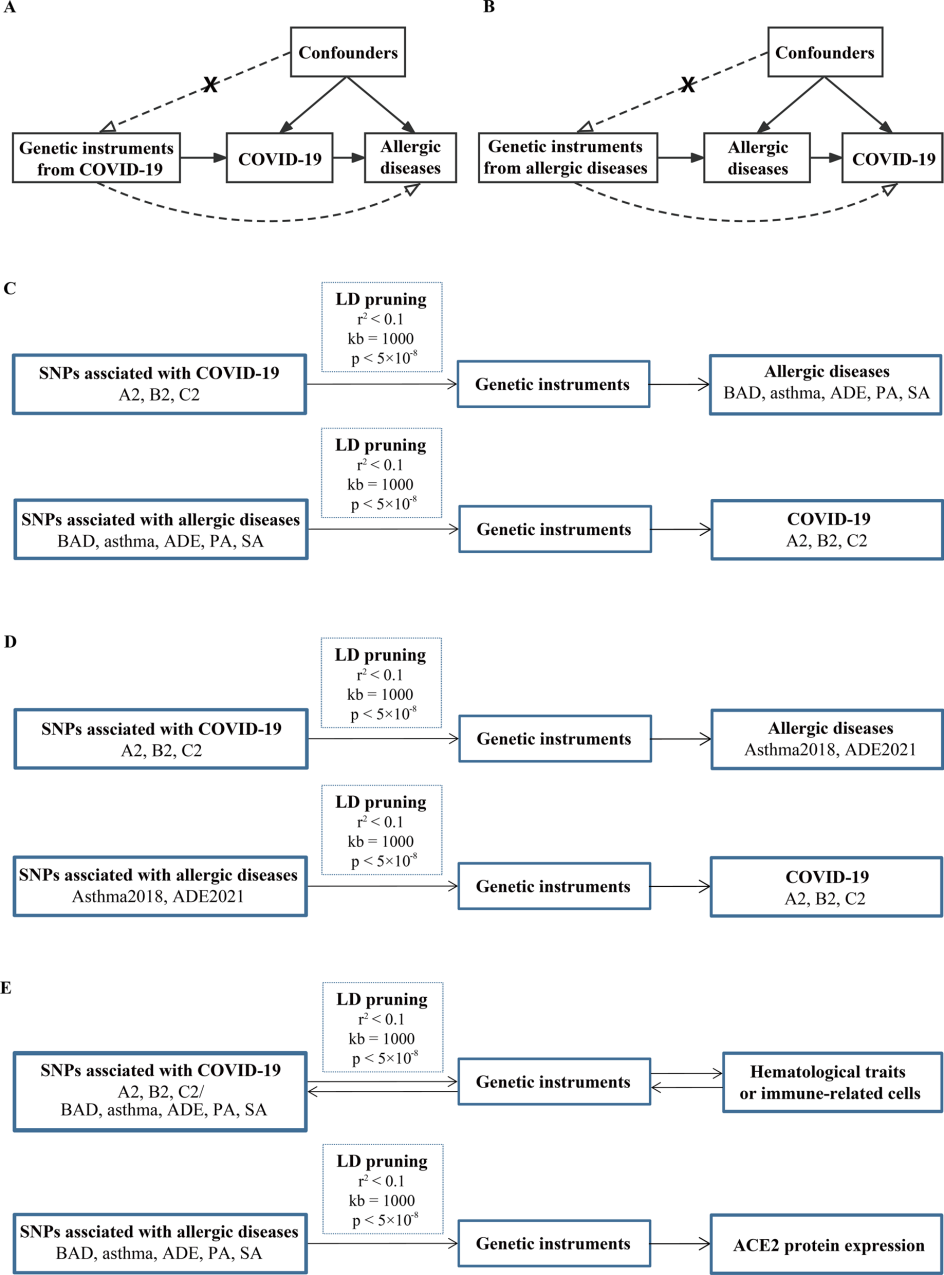
Schematic diagram of principal MR analyses and main flowchart of this study. **A**, **B** Schematic diagram for exploring bidirectional causal correlations between various allergic diseases and COVID-19 phenotypes; **C** exploratory stage for genetically significant correlation between various allergic diseases and COVID-19 phenotypes; **D** validation stage for genetically significant correlation with other available GWAS datasets (Asthma2018 and ADE2021); **E** investigating the underlying molecular mechanisms with publicly available GWAS datasets of hematological traits, immune-related cell counts and ACE2 protein expression level in peripheral blood

The primary MR analyses contained three successive parts. In the first part, we treated each COVID-19 phenotype (A2, B2, and C2) as an exposure and each AD as an outcome to evaluate whether the COVID-19 outbreak would genetically affect the incidence rates of ADs (Fig. 1A, C). Conversely, we considered each type of AD as an exposure and COVID-19 phenotypes as outcomes to identify genetic risk factors for COVID-19 infection/severity (Fig. 1B, C). In the second part, we employed two available independent GWAS datasets (Asthma2018 and ADE2021) to further validate those significant causal correlations identified in the first part (Fig. 1D). Finally, we investigated the molecular mechanisms underlying those significant correlations based on well-known theories of pathogenesis for COVID-19 or ADs (Fig. 1E). In brief, we treated COVID-19 infection/severity or ADs as exposures and peripheral blood hematological traits and count data of immune-related cells as outcomes to evaluate whether COVID-19 infection/severity or ADs played a vital role in affecting hematological traits or immune-related cells. Conversely, we also assessed if there are significant effects of hematological traits or cell counts on COVID-19 infection/severity and ADs. In addition, we also considered various allergic diseases as exposures and peripheral blood ACE2 protein expression as an outcome to explore whether allergic diseases would affect COVID-19 infection/severity by altering ACE2 protein expression in peripheral blood.

### Two-sample MR analysis

First, all the GWAS summary statistics went through multiple pre-processing steps, including transferring genomic coordinates, adding missing but necessary parameters and removing those strong genetically correlated SNPs (*r*^2^ < 0.1, distance = 1000 kb) [37] (see details in Additional file 1: Methods). Independent SNPs with an original *p*-value below 5 × 10^–8^ would be treated as valid instrumental variables. In addition, we also employed MR robust adjusted profile score (RAPS) method, which took into account multiple weak instruments simultaneously using a robust procedure [38].

All two-sample MR analyses were performed using TwoSampleMR (version 0.4.26) package in the R programming language (version 4.2.0). Unlike common applications, we applied the most suitable MR approach in different scenarios. We first employed the MR Egger regression method to determine if there was a horizontal pleiotropy for the genetic instruments and the MR inverse-variance weighted (IVW) method to evaluate if there was an obvious heterogeneity among multiple genetic instruments. Then, the most suitable MR approach was determined in four different scenarios. The fixed-effect IVW method was applied when there was neither horizontal pleiotropy nor significant heterogeneity (scenario 1, Additional file 1: S1). The random-effect IVW method was employed when there was obvious heterogeneity but no horizontal pleiotropy (Additional file 1: S2). The MR Egger method or weighted median was applied when there was an obvious horizontal pleiotropy (Additional file 1: S3). The Wald ratio method was employed when there was only one valid genetic instrument in MR analysis (Additional file 1: S4). With that, only one hypothesis was tested for each pair of exposure and outcome, from which a *p*-value below 0.05 was considered indicative of significant genetic correlations. In terms of effect size in causal correlation when the exposure was a binary trait, the odds ratio (OR) could be roughly considered to represent the likelihood that the outcome would occur when an individual is exposed to one specific condition. In addition, we employed the Steiger directionality test to evaluate if the causal directions between the hypothesized exposures and outcomes are true [39].

## Results

### Selection of genetic instruments

Suitable independent genetic instruments were first selected from all the exposure datasets according to the pre-set criteria (*r*^2^ < 0.1, distance = 1000 kb, *p* < 5 × 10^–8^). In MR analyses that treated ADs as exposures, independent genetic instruments were selected from seven GWAS datasets of ADs (BAD, n = 144; Asthma2020, n = 231; ADE2015, n = 20; PA, n = 5; SA, n = 19; ADE2021, n = 52; and Asthma2018, n = 36) (Tables 1 and 2). For COVID-19 infection and severity, we also obtained 73, 81, and 57 independent instruments for critically ill cases (A2), hospitalized patients (B2), and infection cases (C2), respectively (Additional file 1: Table S2). The detailed number of independent SNPs used in the subsequent MR analyses is also shown in Tables 1 and 2, in which five ADs were treated as exposures and COVID-19 infection/severity were considered as outcomes in each set of analyses.

**Table 1:** Overall MR analyses with strong genetic instruments (*p* < 5E−08) for the causal effects of various allergic diseases on COVID-19 infection/severity

Exposure	Outcome	No. of clumped SNPs^a^	No. of SNPs in MRA^b^	MR *p*-value^c^	MR method^d^	Heterogeneity	Pleiotropy	Directionality
BAD	A2	144	111	**3.26E−02**	IVW (random effects)	Yes	No	True
	B2		115	1.02E−01	IVW (random effects)	Yes	No	True
	C2		118	9.12E−01	IVW (random effects)	Yes	No	True
Asthma2020	A2	231	215	**2.00E−04**	IVW (random effects)	Yes	No	True
	B2		220	**2.01E−02**	IVW (random effects)	Yes	No	True
	C2		223	2.85E−01	IVW (random effects)	Yes	No	True
ADE2015	A2	20	19	5.11E−01	IVW (fixed effects)	No	No	True
	B2		19	*7.98E−02*	IVW (fixed effects)	No	No	True
	C2		20	2.64E−01	IVW (fixed effects)	No	No	True
PA	A2	5	3	2.29E−01	IVW (fixed effects)	No	No	True
	B2		3	1.66E−01	IVW (fixed effects)	No	No	True
	C2		4	**2.66E−02**	IVW (fixed effects)	No	No	True
SA	A2	19	10	**6.73E−03**	IVW (fixed effects)	No	No	True
	B2		10	**1.61E−03**	IVW (fixed effects)	No	No	True
	C2		11	**2.43E−03**	IVW (random effects)	Yes	No	True

^a^No. of clumped SNPs: number of independent genetic SNPs with a p-value < 5 × 10^–8^ after clumping

^b^No. of SNPs in MRA: number of independent genetic SNPs used in the MR analysis for each pair of exposure and outcome

^c^MR *p*-value: p-value of the most suitable MR method

^d^MR method: the most suitable MR analysis used in MR analysis. Bold values indicate that the MR *p*-value is less than 0.05, while italic values indicate that the MR *p*-value is marginally significant

**Table 2:** Overall validated MR analyses with strong genetic instruments (*p* < 5E−08) for causal effects of Asthma2018 on COVID-19 infection/severity

Exposure	Outcome	No. of clumped SNPs^a^	No. of SNPs in MRA^b^	MR *p*-value^c^	MR method^d^	OR	95% LCI	95% UCI
Asthma2018	A2	36	36	**4.22E−03**	IVW (random effects)	0.88	0.80	0.96
				**1.56E−08**	IVW (fixed effects)	0.88	0.84	0.92
				**1.48E−09**	RAPS	0.87	0.83	0.91
	B2		36	**5.61E−03**	IVW (random effects)	0.92	0.87	0.98
				**6.44E−08**	IVW (fixed effects)	0.92	0.89	0.95
				**8.38E−09**	RAPS	0.92	0.89	0.94
	C2		36	*9.23E−02*	IVW (random effects)	0.98	0.95	1.00
				**1.51E−03**	IVW (fixed effects)	0.98	0.96	0.99
				**7.33E−04**	RAPS	0.98	0.96	0.99

*RAPS* Robust Adjusted Profile Score, *OR* odds ratio, *95% LCI* 95% lower confidence interva, *95% UCI* 95% upper confidence interval

^a^No. of clumped SNPs: number of independent genetic SNPs with a p-value < 5 × 10^–8^ after clumping

^b^No. of SNPs in MRA: number of independent genetic SNPs used in the MR analysis for each pair of exposure and outcome

^c^MR *p*-value: p-value of the most suitable MR method

^d^The most suitable MR analysis used in MR analysis. Bold values indicate that the MR *p*-value is less than 0.05, while italic values indicate that the MR *p*-value is marginally significant

### Genetic causal correlations between five types of ADs and COVID-19 infection/severity

**Fig. 2:**
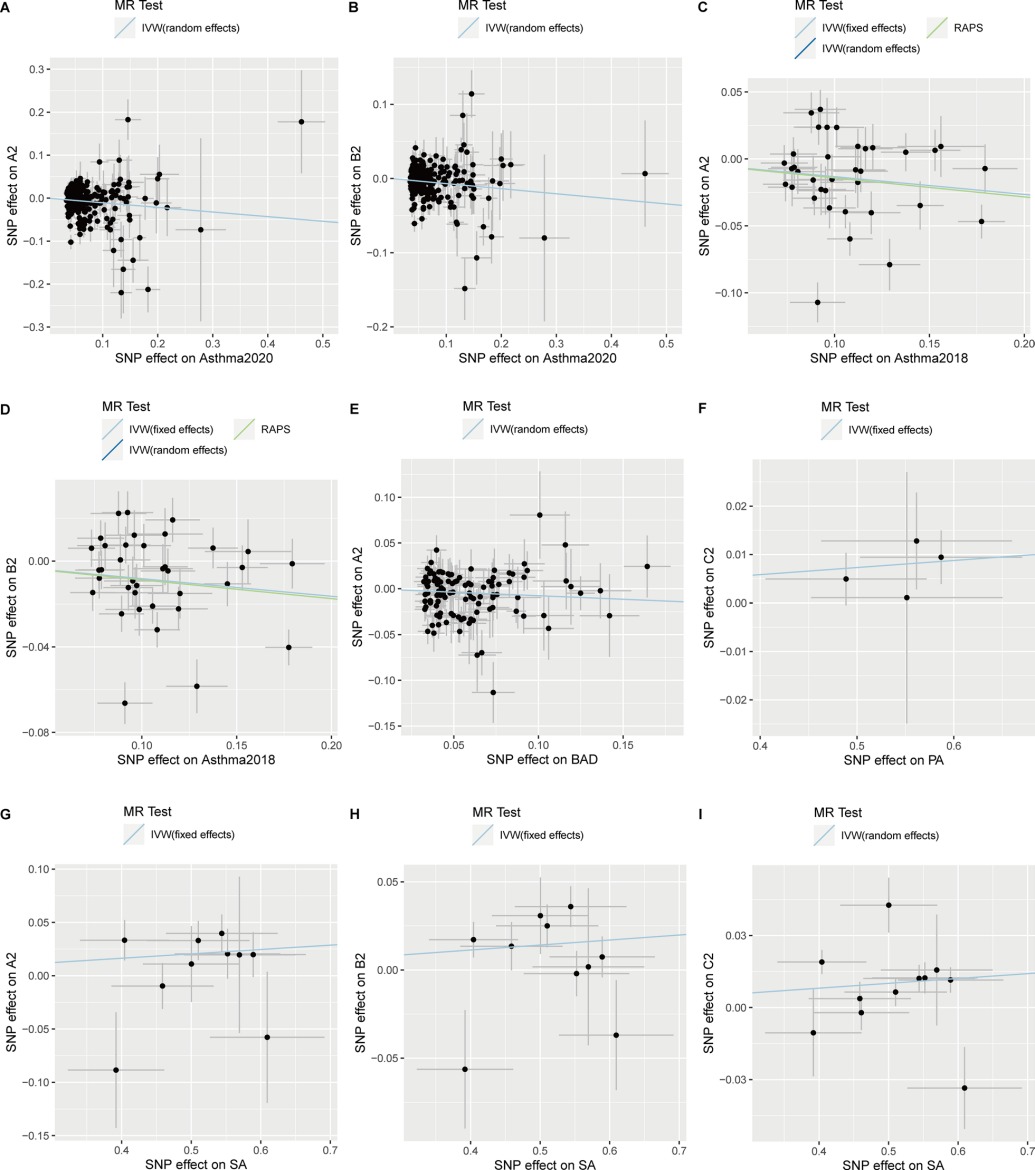
Scatter plots showing significant causal effects of five types of ADs on COVID-19 infection/severity. **A**, **B** Significant causal effects of asthma2020 on COVID-19 A2 and B2; **C**, **D** significant causal effects of asthma2018 on COVID-19 A2 and B2; **E** significant causal effects of broad allergic disease on COVID-19 A2; **F** significant causal effects of peach allergy on COVID-19 C2; **G**–**I** significant causal effects of shrimp allergy on COVID-19 A2, B2, and C2, respectively

In this set of MR analyses, we treated each type of AD as an exposure and COVID-19 infection/severity as an outcome to explore whether there was a significant causal effect of any type of AD on COVID-19 infection/severity. By adopting the most suitable MR method, our MR analyses found that asthma was a causal protective factor for critically ill and hospitalized COVID-19, respectively (OR = 0.90, *p* = 2.00 × 10^–4^; OR = 0.93, *p* = 2.01 × 10^–2^) (Table 1, Fig. 2A, B). Meanwhile, the protective effects of asthma on COVID-19 phenotypes were further confirmed in another GWAS dataset (Asthma2018) with the most suitable IVW (random-effects) MR method, in which asthma was also identified as a protective factor for critically ill and hospitalized COVID-19 (OR = 0.88, *p* = 4.22 × 10^–3^; OR = 0.92, *p* = 5.61 × 10^–3^; IVW(random-effects)) (Table 2, Fig. 2C, D). Besides that, the significant causal effects were also suggested by the other two MR methods, including the IVW (fixed-effects) and RAPS methods (*p* < 6.44 × 10^–8^; Table 2). Regarding the broad allergic disease (BAD), our MR analysis found a significant protective effect of it on critically ill COVID-19 (OR = 0.93, *p* = 3.26 × 10^–2^) (Table 1, Fig. 2E). As for food allergies, our MR analyses observed significantly causal correlations of PA with COVID-19 infection (OR = 1.01, *p* = 2.66 × 10^–2^) and SA with all the three COVID-19 phenotypes (A2: OR = 1.04, *p* = 6.73 × 10^–3^; B2: OR = 1.03, *p* = 1.61 × 10^–3^; C2: OR = 1.02, *p* = 2.43 × 10^–3^) (Table 1, Fig. 2F, G, and I), implying that food allergies may act as risk factors for both COVID-19 infection and severity.

In contrast to the foregoing set, we treated COVID-19 infection/severity as exposures and each type of AD as an outcome to evaluate if the prevalence of ADs would be affected by COVID-19 infection/severity. With the most suitable MR method, our MR analyses only observed a marginal negative correlation between COVID-19 infection (C2) and PA (Additional file 1: Table S2). However, the acting direction of the marginal correlation was found to be false by the directionality test. Except that, we did not find any other significant causal effect of COVID-19 infection/severity on ADs (Additional file 1: Table S2).

### Genetic causal correlations between COVID-19 infection/severity and peripheral blood immune-related cells and hematological traits

**Fig. 3:**
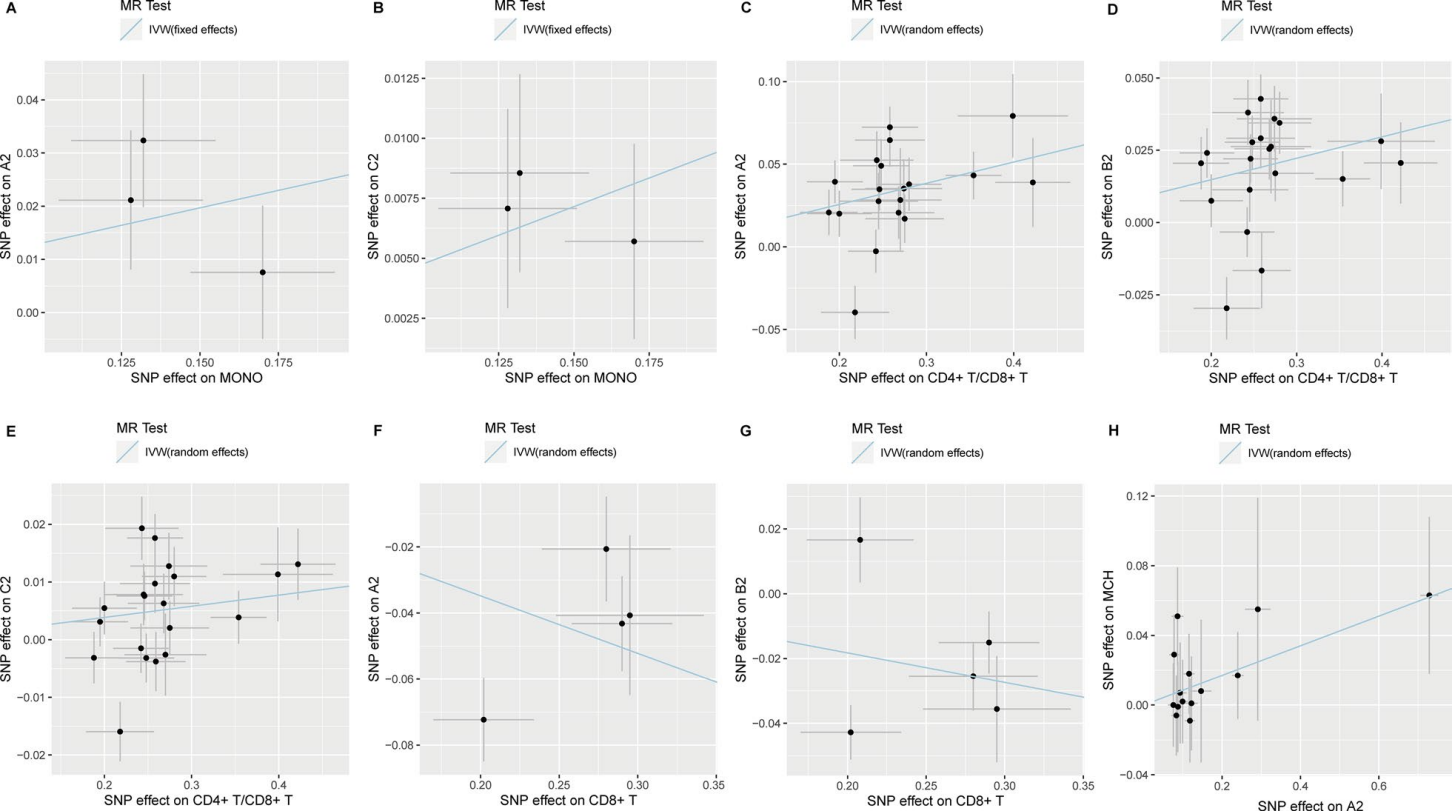
Scatter plots showing significant causal effects of peripheral blood hematological traits and immune-related cells on COVID-19 infection/severity. **A**, **B** Significant causal effects of monocytes on COVID-19 A2 and B2; **C**–**E** significant causal effects of the ratio of CD4^+^ T/CD8^+^ T cells on COVID-19 A2, B2, and C2; **F**, **G** significant causal effects of CD8^+^ T cells on COVID-19 A2 and B2; **H** significant causal effects of COVID-19 A2 on mean cell hemoglobin. Scatter plots for CD4^+^ T and CD56^+^ natural killer cells could not be drawn as only one valid SNP in MR analysis

We aimed to investigate the molecular mechanism underlying the causal correlations observed between COVID-19 phenotypes and various ADs. In this set of MR analyses, we first treated 13 types of hematological traits and 6 kinds of immune-related cell counts as exposures and three COVID-19 phenotypes as outcomes to explore whether the immune-related traits/cells in peripheral blood would significantly affect COVID-19 infection/severity. The most suitable MR methods observed a causal risk effect of monocyte counts on critically ill COVID-19 (OR = 1.14, *p* = 9.39 × 10^–3^) and COVID-19 infection (OR = 1.05, *p* = 3.63 × 10^–3^), respectively (Table 3) (Fig. 3A, B). In addition, the ratio of CD4^+^ and CD8^+^ T cells and the CD4^+^ T cell counts were found to be risk factors for all COVID-19 phenotypes (CD4^+^ T/8^+^ T: OR = 1.02–1.14, *p* = 7.21 × 10^–9^–4.63 × 10^–3^_,_ Fig. 3C–E; CD4^+^ T: OR = 1.07–1.31, *p* = 3.17 × 10^–4^–3.42 × 10^–3^), while CD8^+^ T cell counts had a protective causal effect on critically ill (OR = 0.84, *p* = 3.83 × 10^–3^) and hospitalized COVID-19 cases (OR = 0.91, *p* = 2.17 × 10^–2^), respectively (Table 3) (Fig. 3F, G). Besides that, the CD56^+^ natural killer cell counts showed a causal risk effect on hospitalized COVID-19 cases (OR = 1.12, *p* = 2.83 × 10^–3^). Notably, all this set of MR analyses were indicated to be true in the directionality tests (Table 3).

**Table 3:** Overall MR analyses with strong genetic instruments (*p* < 5E−08) for causal effects of peripheral blood hematological traits and count data of immune-related cells on COVID-19 infection/severity

Exposure	Outcome	No. of clumped SNPs^a^	No. of SNPs in MRA^b^	MR *p*-value^c^	MR method^d^	Heterogeneity	Pleiotropy	Directionality
Immune-related cells								
CD4^+^ T/CD8^+^T	A2	21	19	**7.21E−09**	IVW (random effects)	Yes	No	True
	B2		20	**5.36E−07**	IVW (random effects)	Yes	No	True
	C2		20	**4.63E−03**	IVW (random effects)	Yes	No	True
CD4^+^ T	A2	1	1	**3.17E−04**	Wald ratio	–	–	True
	B2		1	**3.42E−03**	Wald ratio	–	–	True
	C2		1	**3.38E−03**	Wald ratio	–	–	True
CD56^+^ NK	A2	1	1	1.66E−01	Wald ratio	–	–	True
	B2		1	**2.83E−03**	Wald ratio	–	–	True
	C2		1	3.85E−01	Wald ratio	–	–	True
CD8^+^ T	A2	5	4	**3.83E−03**	IVW (random effects)	Yes	No	True
	B2		5	**2.17E−02**	IVW (random effects)	Yes	No	True
	C2		5	*8.88E−02*	IVW (random effects)	Yes	No	True
Hematological traits								
HT	A2		1	4.92E−01	Wald ratio	–	–	True
	B2	1	1	2.14E−01	Wald ratio	–	–	True
	C2		1	4.42E−01	Wald ratio	–	–	True
LYMPH	A2	1	1	7.81E−01	Wald ratio	–	–	True
	B2		1	6.96E−01	Wald ratio	–	–	True
	C2		1	6.18E−01	Wald ratio	–	–	True
MCH	A2	4	3	4.86E−01	IVW (fixed effects)	No	No	True
	B2		3	4.40E−01	IVW (fixed effects)	No	No	True
	C2		3	2.50E−01	IVW (fixed effects)	No	No	True
MCV	A2	5	5	3.58E−01	IVW (fixed effects)	No	No	True
	B2		5	4.92E−01	IVW (fixed effects)	No	No	True
	C2		5	4.37E−01	IVW (fixed effects)	No	No	True
MONO	A2	3	3	**9.39E−03**	IVW (fixed effects)	No	No	True
	B2		3	*5.11E−02*	IVW (fixed effects)	No	No	True
	C2		3	**3.63E−03**	IVW (fixed effects)	No	No	True
PLT	A2	1	1	*7.71E−02*	Wald ratio	–	–	True
	B2		1	7.89E−01	Wald ratio	–	–	True
	C2		1	8.95E−01	Wald ratio	–	–	True
RBC	A2	1	1	1.78E−01	Wald ratio	–	–	True
	B2		1	7.21E−01	Wald ratio	–	–	True
	C2		1	8.12E−01	Wald ratio	–	–	True

–: The corresponding analysis is not available

^a^No. of clumped SNPs: number of independent genetic SNPs with a p-value < 5 × 10^–8^ after clumping

^b^No. of SNPs in MRA: number of independent genetic SNPs used in the MR analysis for each pair of exposure and outcome

^c^MR *p*-value: p-value of the most suitable MR method

^d^The most suitable MR analysis used in MR analysis. Bold values indicate that the MR *p*-value is less than 0.05, while italic values indicate that the MR *p*-value is marginally significant

Conversely, we treated three kinds of COVID-19 phenotypes as exposures and the 19 immune-related traits/cells as outcomes and found some significant correlations (Additional file 1: Table S3). However, most of the directionality tests in MR analyses were found to be false, except for the mean cell hemoglobin (MCH). The critically ill COVID-19 cases showed a marginal significant association with an increased MCH level in peripheral blood (OR = 1.09, *p* = 4.79 × 10^–2^) (Additional file 1: Table S3) (Fig. 3H).

### Genetic causal correlations between five ADs and peripheral blood hematological traits, immune-related cell counts and ACE2 protein expression

**Fig. 4:**
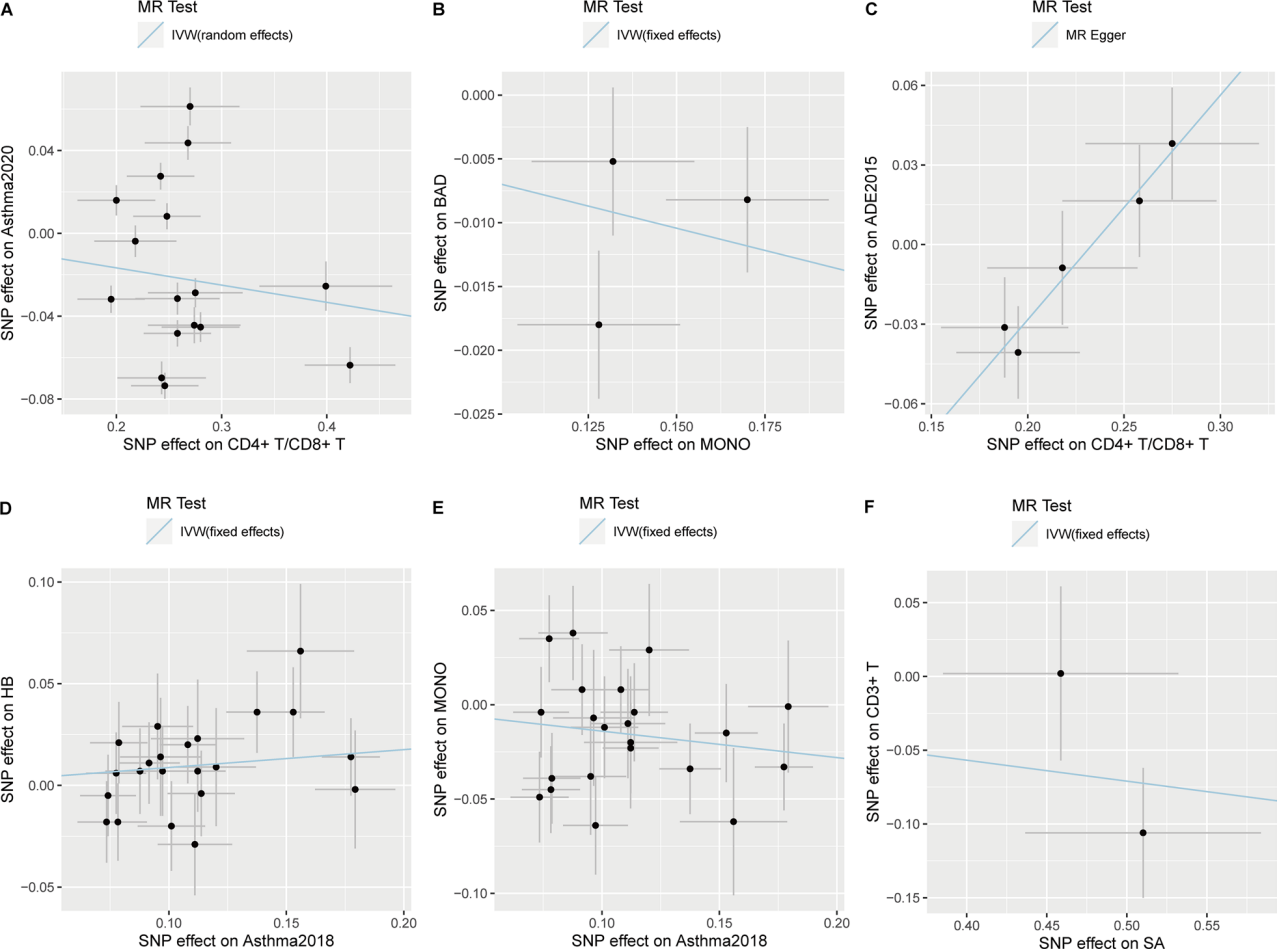
Scatter plots showing significant causal correlations between various allergic diseases and peripheral blood immune-related cells and hematological traits. **A**, **C** Significant causal effects of the ratio of CD4^+^ T/CD8^+^ T cells on asthma and allergic dermatitis, respectively; **B** significant causal effects of monocytes on BAD; **D**, **E** significant causal effects of asthma2018 on total blood hemoglobin and monocytes, respectively; **F** significant causal effects of shrimp allergy on CD3^+^ T cells. Scatter plots for CD4^+^ T and lymphocyte cells could not be drawn as only one valid SNP in MR analysis

In this set of MR analyses, we first treated 13 types of hematological traits and 6 kinds of immune-related cell counts as exposures and five types of ADs as outcomes to explore if the immune-related traits in peripheral blood would affect the occurrence of various ADs. With the most suitable MR methods, we observed that two kinds of immune-related cells had protective causal effects on asthma (CD4^+^ T: OR = 0.78, *p* = 1.73 × 10^–13^; CD4^+^ T/CD8^+^ T: OR = 0.92, *p* = 2.42 × 10^–02^) (Table 4 and Fig. 4A). Besides that, the lymphocyte cell counts also showed a protective effect on asthma (OR = 0.68, *p* = 5.23 × 10^–14^). Regarding the broad allergic disease (BAD), CD4^+^ T cells and monocyte cells had significant causal effects on it (*p* = 3.90 × 10^–4^ and *p* = 2.46 × 10^–3^) (Fig. 4B). Besides that, the CD4^+^ T cell showed a risk causal effect on PA (OR = 3.92, *p* = 2.74 × 10^–4^) and the ratio of CD4^+^ T/CD8^+^ T also had a risk effect on atopic dermatitis (OR = 2.33, *p* = 4.70 × 10^–2^) (Fig. 4C). The directionality tests confirmed that all the directions of MR analyses were true. Conversely, we considered five types of ADs as exposures and found significant causal correlations of asthma with total blood hemoglobin and monocytes, respectively (*p* = 3.66 × 10^–2^ and *p* = 5.46 × 10^–3^) (Additional file 1: Table S3) (Fig. 4D, E). In addition, shrimp allergy had a significant causal effect on CD3^+^ T cells (*p* = 4.75 × 10^–2^, Fig. 4F) and multiple hematological traits, including lymphocytes, monocytes, neutrophils and white blood cells (*p* = 6.30 × 10^–6^–1.85 × 10^–2^) (Additional file 1: Table S3). Although our MR analyses observed other significant correlations in this set of MR analyses, the directionality tests identified the directions of these correlations were false.

**Table 4:** Overall MR analyses with strong genetic instruments (*p* < 5E−08) for causal effects of peripheral blood hematological traits and count data of immune-related cells on five types of ADs

Exposure	Outcome	No. of clumped SNPs^a^	No. of SNPs in MRA^b^	MR *p*-value^c^	MR method^d^	Heterogeneity	Pleiotropy	Directionality
Immune-related cells								
CD4^+^ T	BAD	1	1	**3.90E−04**	Wald ratio	–	–	True
	Asthma2020		1	**1.73E−13**	Wald ratio	–	–	True
	PA		1	**2.74E−04**	Wald ratio	–	–	True
	SA		1	2.85E−01	Wald ratio	–	–	True
CD4^+^ T/CD8^+^ T	BAD	21	20	9.69E−01	IVW (random effects)	Yes	No	True
	Asthma2020		16	**2.42E−02**	IVW (random effects)	Yes	No	True
	ADE2015		5	**4.70E−02**	MR Egger	Yes	Yes	True
	PA		16	1.46E−01	IVW (random effects)	Yes	No	True
	SA		16	7.79E−01	IVW (random effects)	Yes	No	True
CD56^+^ NK	BAD	1	1	5.49E−01	Wald ratio	–	–	True
	Asthma2020		1	2.66E−01	Wald ratio	–	–	True
	ADE2015		1	7.16E−01	Wald ratio	–	–	True
CD8^+^ T	BAD	5	5	3.01E−01	IVW (random effects)	Yes	No	True
	Asthma2020		3	7.31E−01	IVW(random effects)	Yes	No	True
	PA		4	7.57E−01	IVW (fixed effects)	No	No	True
	SA		4	5.44E−01	IVW (random effects)	Yes	No	True
Hematological traits								
HT	BAD	1	1	3.00E−01	Wald ratio	–	–	True
	Asthma2020		1	3.58E−01	Wald ratio	–	–	True
	ADE2015		1	3.83E−01	Wald ratio	–	–	True
LYMPH	BAD	1	1	5.50E−01	Wald ratio	–	–	True
	Asthma2020		1	**5.23E−14**	Wald ratio	–	–	True
MCH	BAD	4	3	4.58E−01	IVW (random effects)	Yes	No	True
	Asthma2020		3	4.02E−01	IVW (fixed effects)	No	No	True
	ADE2015		3	4.74E−01	IVW (fixed effects)	No	No	True
	PA		1	6.01E−01	Wald ratio	–	–	True
	SA		1	7.82E−01	Wald ratio	–	–	True
MCV	BAD	5	5	1.88E−01	IVW (fixed effects)	No	No	True
	Asthma2020		4	2.00E−01	IVW (fixed effects)	No	No	True
	ADE2015		5	3.63E−01	IVW (fixed effects)	No	No	True
	PA		1	6.01E−01	Wald ratio	–	–	True
	SA		1	7.82E−01	Wald ratio	–	–	True
MONO	BAD	3	3	**2.46E−03**	IVW (fixed effects)	No	No	True
	Asthma2020		3	3.23E−01	IVW (fixed effects)	No	No	True
	ADE2015		3	8.52E−01	IVW (fixed effects)	No	No	True
PLT	BAD	1	1	8.80E−01	Wald ratio	–	–	True
	Asthma2020		1	4.11E−01	Wald ratio	–	–	True
	ADE2015		1	4.90E−01	Wald ratio	–	–	True
RBC	BAD	1	1	8.03E−01	Wald ratio	–	–	True
	Asthma2020		1	5.24E−01	Wald ratio	–	–	True
	ADE2015		1	9.20E−01	Wald ratio	–	–	True

–: The corresponding analysis is not available

^a^No. of clumped SNPs: number of independent genetic SNPs with a p-value < 5 × 10^–8^ after clumping

^b^No. of SNPs in MRA: number of independent genetic SNPs used in the MR analysis for each pair of exposure and outcome

^c^MR *p*-value: p-value of the most suitable MR method

^d^The most suitable MR analysis used in MR analysis. Bold values indicate that the MR *p*-value is less than 0.05

As for ACE2 protein expression, we treated five types of ADs as exposures and peripheral blood ACE2 protein expression level from two GWAS datasets as outcomes to investigate if causal effects of ADs on COVID-19 phenotypes were mediated by the ACE2 expression level. Employing 220 strong genetic instruments, our MR analyses found a marginal correlation between asthma and ACE2 protein expression level (OR = 1.02, *p* = 5.74 × 10^–2^) (Additional file 1: Table S4). In addition, we also observed a marginal negative correlation of shrimp allergy with ACE2 protein expression (OR = 0.87, *p* = 8.93 × 10^–2^) (Additional file 1: Table S4). But beyond that, the MR analyses did not find any other significant/marginal correlation between various ADs and ACE2 protein expression level.

## Discussion

In the present study, our systematic two-sample and bidirectional MR analysis process identified unidirectional causal effects of various ADs, particularly for a protective causal effect of asthma on COVID-19 infection/severity, but the reverse is not true. As for the underlying molecular mechanisms, the unidirectional causal effects may be mediated by abnormal fluctuations of immune-related cells and hematology traits in peripheral blood, such as CD4^+^ T, CD8^+^ T and monocyte cells. The change of ACE2 protein expression level might also play a non-negligible role on the causal correlations. The two-sample MR approach is a relatively well-established technique for evaluating causal correlations for diseases/traits of interest using GWAS summary statistics [40].

Here we utilized MR approach to show unidirectional causal effects of various ADs on COVID-19 phenotypes, including a protective causal effect of asthma on COVID-19, but not vice versa. These unidirectional causal effects may be mediated by fluctuations in amounts of immune-related cells in the peripheral blood, including CD4^+^ and CD8^+^ T cells as well as monocytes, and by other hematological traits. The circulating levels of ACE2 protein might also contribute to observed causal correlations.

### Strong protective causal effects of asthma on COVID-19 infection/severity

Using the most suitable MR method, our MR analyses suggested that asthma was a causal protective factor for critically ill and hospitalized COVID-19 cases (OR < 0.93), which was further validated by another independent asthma GWAS dataset with multiple MR methods (OR, 0.87–0.98). The validation MR analysis confirmed a solid, negative causal correlation of asthma with severe course of COVID-19 (OR, 0.87–0.92), suggesting asthma patients may experience less severe symptoms following infection with COVID-19. Although it was commonly taken for granted that asthma patients showed increased susceptibility to COVID-19, or develop a disease of greater severity [41], the results of conventional observational studies either do not support this this presumption or contradict each other [6, 8, 10, 42]. Two previous unidirectional MR analyses also reported conclusions that were opposite to each other, possibly due to use of different GWAS datasets [43, 44], and, therefore, varying sets of independent genetic instruments. Moreover, lacking validation step in an independent dataset would to some extent decrease the reliability of outcome. Our MR analyses did not only include a validation step but also utilized the largest GWAS dataset to date for the analysis [27], which contained the largest number of independent genetic instruments to infer the correlation and generated highly promising results of protective causal effects.

### Immune-related cells mediated protective causal effects of asthma on COVID-19 infection/severity

Our MR analyses implied that the protective causal effects of asthma on COVID-19 infection/severity were mediated by abnormal change of multiple immune-related cells, mainly including the CD4^+^ T cells, CD8^+^ T cells, the ratio of CD4^+^ T/CD8^+^ T and the monocyte cells in peripheral blood. The ratio of CD4^+^ T/CD8^+^ T cells in the peripheral blood of healthy adults and mice is approximately 2:1, and breaking the balance usually indicates diseases relating to immunodeficiency induced by an impaired immune system [45–48]. Recently Huang et al*.* performed a systematic meta-analysis and found that COVID-19 patients showed significantly declined count of B cells, natural killer cells and total lymphocytes [49]. Notably, both the CD4^+^ and CD8^+^ T cells had a far greater extent of decrease. Moreover, a study of 60 COVID-19 patients suggested that the decreased CD8+ T cells and the increased ratio of CD4^+^/CD8^+^ T cells were associated with poor treatment efficacy [50], which provided strong evidence to our findings about the positive genetic causal effect of an increased ratio and the inverse causal effect of a decreased CD8^+^ T cell count. Patients with asthma also showed an increased ratio and a decreased CD8^+^ T cell count in peripheral blood compared to healthy controls [51]. After 7-day antigen stimulation, the ratio would have a significant increase in atopic dermatitis syndrome patient’s peripheral blood mononuclear cells (PBMCs) as compared with healthy control’s cells [52].

Regarding the monocyte cells in peripheral blood, Ren et al*.* applied single-cell RNA sequencing to 284 samples from 196 COVID-19 patients and reported that the percentage of CD14^+^ monocytes in PBMCs was significantly elevated in COVID-19 patients, especially in COVID-19 hospitalized and critically ill patients [53]. Meanwhile, subtypes of macrophages and monocytes had the highest cytokine and inflammatory scores in the severe COVID-19 samples, implying that these cell subtypes might be the major sources driving the inflammatory storm in lung tissue. The main manifestation of COVID-19 severity was severe acute respiratory distress syndrome, which was mainly caused by pneumonia, sepsis, or pulmonary aspiration induced by a cytokine storm. During a cytokine storm, immune and non-immune cells would release a large number of pro-inflammatory cytokines, including IL-6, tumor necrosis factor-α (TNF-α), and chemokines, which would cause substantial damage to the host immune response and further induce various ADs such as allergic asthma [54, 55]. These findings are consistent with our present results about a positive causal correlation between monocytes and COVID-19 cases, particularly in those severe patients.

The protective effects were also partially explained by the MR analyses of asthma and ACE2 protein expression using two different MR methods, which showed that asthma was marginally correlated with the protein expression of soluble ACE2 in peripheral blood. The soluble ACE2 has a protective effect against virus-induced lung injury by increasing the amount of angiotensin 1–7, which produces local vasodilation, thereby reducing blood pressure [56, 57]. In contrast, the surface bound ACE2 is the main receptor for COVID-19 virus to invade into cells that increasing the incidence rate of COVID-19 infection [36, 58]. Several previous studies have suggested that allergic sensitization in asthma patients is associated with a low surface bound ACE2 expression in sputum cells in the upper and lower respiratory tracts [4, 59]. In addition, the *ACE2* gene is less active in asthma patients and probably limits viral entry into the respiratory epithelium [60]. Moreover, it was reported that epithelial surface bound ACE2 expression is inversely associated with the levels of Th2 cytokines (IL-4, IL-5, and IL-13) [61] that could alleviate the viral-induced release of interferons and downregulate the cytokine storm typical of increasing COVID-19 severity [62].

### Potential causal effects of BAD and food allergies on COVID-19 infection/severity

In addition to asthma, the present MR analyses also found a potential protective causal effect of BAD on critically ill COVID-19 (OR = 0.93). It is worth noting that the BAD is a combination of asthma, hay fever and atopic dermatitis. Because of the negative causal correlations between atopic dermatitis and COVID-19 infection/severity in our MR analyses, the causal effect is more likely driven by the protective causal effect of asthma. The exact effect and interaction roles are worthy to be further investigated if GWAS summary statistics for the three types of individual allergic diseases are publicly available. Besides that, we identified risk causal effects of food allergies on COVID-19 phenotypes (OR > 1.01), particularly for shrimp allergy on all three COVID-19 phenotypes (OR > 1.02). Although the findings could not be further validated due to a lack of another available GWAS dataset, our MR analyses partially explained the molecular mechanism underlying the causal effects. The shrimp allergy not only showed significant effects on multiple immune-related cells and hematology traits but also have a marginal association with ACE2 protein expression level. The close relationships between food allergies and immune-related cells obtained supports from one previous study [63]. In cord blood, infants who developed food allergies showed a higher ratio of monocyte/CD4^+^ T cells, which would secrete higher amounts of inflammatory cytokines, such as IL-1β, IL-6, and TNF-α that further suppressed IL-2 expression by CD4^+^ T cells. With the decrease of IL-2, multiple inflammatory cytokines further decreased the number of activated natural regulatory T cells and generated an IL-4 expression non-classical Th2 phenotype, which subsequently induced a series of allergic reactions. Our MR analyses for the first time found potentially causal correlations between BAD and food allergies with COVID-19 phenotypes, calling more attention to other types of ADs.

### Limitations

Several limitations in the present study should be elucidated. First, we could not obtain another GWAS dataset for the two kinds of food allergies, which weakened the reliability of causal correlations between food allergies and COVID-19 phenotypes. Although GWAS analyses of food allergies were performed in eastern Asian populations, the analyses comprised multiple steps to control for population stratification to minimize its impact on the final results as much as possible. In addition, our MR analyses identified a series of significant causal effects of immune-related cells on ADs and COVID-19 phenotypes, but a part of analyses only depended on one valid genetic variant, which is likely to decrease the reliability of results and the acting direction. However, the Wald ratio method was designed to estimate genetic correlation of diseases/traits of interest and could ensure the reliability of outcome in this scenario [64]. Finally, although we obtained two GWAS datasets of ACE2 protein expression that could directly reflect the effective level of the protein that exerts a biological function in peripheral blood tissue, it only indirectly reflected ACE2 protein expression in lung or respiratory tract tissue. Notably, our MR analyses about the potential correlations of asthma on ACE2 protein expression were supported by previous observational studies that conducted in the respiratory tract [4, 59].

## Conclusions

Our systematic two-sample and bidirectional MR analyses consistently indicated the existence of a unidirectional protective causal effect of asthma on COVID-19 infection/severity, which was confirmed by another GWAS dataset and could be further explained by the underlying molecular mechanisms. In brief, asthma symptoms were causally associated with abnormal fluctuations of immune-related cells and hematological traits in peripheral blood. Our findings also implied potential causal effects of food allergies on COVID-19 phenotypes, which require further testing in observational or fundamental studies. The findings of our MR analyses suggest that in addition to focusing on the widely studied asthma for infection/severity to develop effective preventive measures for asthma patients, we should also pay attention to the other allergic diseases, which may also induced by the dysfunction of host’s immune responses.

### Supplementary Information

**Additional file 1:** Supplementary Methods: Pre-processing of GWAS summary statistics. Supplementary Results: Table S1. Basic characteristics of GWAS summary statistics for COVID-19, various types of allergic diseases, ACE2 protein expression, and peripheral blood hematological traits and immune-related cells. Table S2. Overall MR analyses with strong genetic instruments (*p* < 5E-08)  for causal effects of COVID-19 infection/severity on five types of allergic diseases. Table S3. Overall MR analyses with strong genetic instruments (*p* < 5E-08) for causal effects of COVID-19 infection/severity or five types of ADs on peripheral blood hematological traits and immune-related cells. Table S4. MR analyses with strong genetic instruments (*p* < 5E-08) for causal effects of asthma and shrimp allergy on peripheral blood ACE2 protein expression level.

## Data Availability

All data generated or analyzed during this study are included in this published article and its Additional files.

## Abbreviations

ADs: Allergic diseases
BAD: Board allergic disease
ADE: Allergic dermatitis
SA: Shrimp allergy
PA: Peach allergy
A2: COVID-19 critical ill cases
B2: COVID-19 hospitalized patients
C2: COVID-19 infection cases
MR: Mendelian randomization
GWAS: Genome-wide association study
OR: Odds ratio
RCT: Randomized controlled trial
SNP: Single nucleotide polymorphism
ACE2: Angiotensin-converting enzyme 2
HB: Hemoglobin
MCV: Mean corpuscular volume
PLT: Platelet
HT: Hematocrit
MCH: Mean cell hemoglobin
NEUT: Neutrophil
MONO: Monocyte
EOS: Eosinophil
BASO: Basophil
LYMPH: Lymphocyte
RAPS: Robust adjusted profile score
IVW: Inverse-variance weighted
