# Supplementary material for: A systematic two-sample and bidirectional MR process highlights a unidirectional genetic causal effect of allergic diseases on COVID-19 infection/severity
Source: J Transl Med. 2024 Jan 23;22:94. doi: 10.1186/s12967-024-04887-4 (PMC10804553; doi:10.1186/s12967-024-04887-4)
Supplement: Supplementary file 1 — Additional file 1: Supplementary Methods: Pre-processing of GWAS summary statistics. Supplementary Results: Table S1. Basic characteristics of GWAS summary statistics for COVID-19, various types of allergic diseases, ACE2 protein expression, and peripheral blood hematological traits and immune-related cells. Table S2. Overall MR analyses with strong genetic instruments (p < 5E-08) for causal effects of COVID-19 infection/severity on five types of allergic diseases. Table S3. Overall MR analyses with strong genetic instruments (p < 5E-08) for causal effects of COVID-19 infection/severity or five types of ADs on peripheral blood hematological traits and immune-related cells. Table S4. MR analyses with strong genetic instruments (p < 5E-08) for causal effects of asthma and shrimp allergy on peripheral blood ACE2 protein expression level. [file 12967_2024_4887_MOESM1_ESM.docx]

**Additional file 1**

**Supplementary Methods**

*Pre-processing of GWAS summary statistics*

First, we employed the LiftOver program (University of California Santa Cruz, CA, USA) to transfer the genomic positions into Ensembl GRCh37 release 108 for all the GWAS summary statistics. Afterward, several missing but necessary parameters were calculated using different approaches. Summary data without beta or standard error values were calculated using equations (1) and (2), respectively. The summary data of asthma (2020) only included the minor allele frequency (MAF) for the original two datasets individually (UK Biobank and Iceland) instead of the whole dataset. The MAF data for the whole dataset were thus calculated using equation (3). Besides, more than half of the summary datasets lacked data regarding the necessary non-effect alleles, which would be added based on the reference genome (GRCh37). After adding the missing parameters, we further removed SNPs with a strong genetic correlation in the linkage disequilibrium block (*r*^2^ <0.1, distance=1000 kb) using the PLINK program (version 1.90). The correlations between SNPs were derived from the 1000 Genomes European or eastern Asian samples. SNPs with an original *p*-value below 5×10^-8^ would be treated as valid instrumental variables. In addition, we used a more relaxed *p*-value threshold (5×10^-3^) to select genetic variants for the MR robust adjusted profile score (RAPS) method, which took into account multiple weak instruments simultaneously using a robust procedure.

| $\mathrm{beta}=log(OR)$ 1 | |  |
| --- | --- | --- |
| $\mathrm{se}=\frac{log(\mathrm{OR})}{qnorm(\frac{P}{2})}, se=\frac{\sqrt{\mathrm{beta}^{2}}}{qchisq(P)}$ 2 |  |  |
| $f_{meta}=\frac{{A1}_{\mathrm{IS}}+ {A1}_{\mathrm{UKBB}}}{2(N_{\mathrm{IS}}+N_{\mathrm{UKBB}})}$ 3 | | |

OR: odds ratio for effect allele in the summary data; *P*: original *p*-value from the GWAS analysis. A1_IS_ and A1_UKBB_: number of effect alleles in Iceland dataset and UKBB dataset; *f_IS_* and *f_UKBB_*: MAF value in the Iceland dataset and UKBB dataset; N_IS_ and N_UKBB_: sample size in the Iceland dataset and UKBB dataset; *f_meta_*: MAF value in the combined dataset; se, standard error.

**Supplementary Results**

**Table S1.** Basic characteristics of GWAS summary statistics for COVID-19, various types of allergic diseases, ACE2 protein expression, and peripheral blood hematological traits and immune-related cells.

| **Phenotype** | **Abbreviation** | **Cases** | **Controls** | **N** | **No. of SNPs** | **PMID** |
| --- | --- | --- | --- | --- | --- | --- |
| COVID-19-A2 | A2 | 18,152 | 1,145,546 | 1,163,698 | 11,707,239 | NA |
| COVID-19-B2 | B2 | 44,986 | 2,356,386 | 2,401,372 | 12,018,444 | NA |
| COVID-19-C2 | C2 | 159,840 | 2,782,977 | 2,942,817 | 14,328,058 | NA |
| 2017 Board allergic disease | BAD | 96,794 | 145,775 | 242,569 | 8,307,659 | 29083406 |
| 2020 Asthma | Asthma2020 | 69,189 | 702,199 | 771,388 | 9,791,651 | 31959851 |
| 2018 Asthma | Asthma2018 | 23,948 | 118,538 | 142,486 | 2,001,280 | 29273806 |
| 2021 Atopic dermatitis | ADE2021 | 22,474 | 774,187 | 796,661 | 15,601,007 | 34454985 |
| 2015 Atopic dermatitis | ADE2015 | 21,399 | 95,464 | 116,863 | 11,296,420 | 26482879 |
| Peach allergy | PA | 320 | 8,350 | 8,670 | 580,874 | 29348432 |
| Shrimp allergy | SA | 539 | 8,350 | 8,889 | 581,394 | 29348432 |
| 13 Hematological traits | HB, RBC, MCV, PLT, WBC,  HT, MCH, MCHC, NEUT,  MONO, EOS, BASO, LYMPH | NA | NA | 6,015 | 1,860,149 | 19853236 |
| 6 Immune-related cells | CD3^+^ T, CD4^+^ T, CD8^+^ T,  CD19^+^ B, CD56^+^ NK,  CD4^+^ T:CD8^+^ T | NA | NA | 2,538 | 2,337,598 | 20045101 |
| 2017 ACE2 in plasma | ACE2-2017 | NA | NA | 1,000 | 507,099 | 28240269 |
| 2022 ACE2 in plasma | ACE2-2022 | NA | NA | 28,204 | 17,449,412 | 35387486 |

NA, not available; HB, total blood hemoglobin; RBC, red blood cell count; MCV, mean corpuscular volume; PLT, platelet count; WBC, white blood cell count; HT, hematocrit; MCH, mean cell hemoglobin; MCHC, mean corpuscular hemoglobin concentration; NEUT, neutrophil counts; MONO, monocyte counts; EOS, eosinophil counts; BASO, basophil counts; LYMPH, lymphocyte counts; CD3^+^ T, CD3^+^ T cells; CD4^+^ T, CD4^+^ T cells; CD8^+^ T, CD8^+^ T cells; CD19^+^ B, CD19^+^ B cells; CD56^+^ NK, natural killer cells; CD4^+^ T:CD8^+^ T, ratio of CD4^+^ T cells: CD8^+^ T cells.

**Table S2. Overall MR analyses with strong genetic instruments (*p*<5E-08) for causal effects of COVID-19 infection/severity on five types of allergic diseases.**

| **Exposure** | **Outcome** | **No. of clumped SNPs ^a^** | **No. of SNPs in MRA ^b^** | **MR**  ***p*-value ^c^** | **MR**  **Method ^d^** | **Heterogeneity** | **Pleiotropy** | **Directionality** |
| --- | --- | --- | --- | --- | --- | --- | --- | --- |
| A2 | BAD | 73 | 63 | 7.41E-01 | IVW(random effects) | Yes | No | True |
|  | Asthma2020 |  | 64 | 2.59E-01 | IVW(random effects) | Yes | No | True |
|  | ADE2015 |  | 66 | 1.13E-01 | IVW(random effects) | Yes | No | True |
|  | PA |  | 10 | 2.76E-01 | IVW(random effects) | Yes | No | False |
|  | SA |  | 10 | 8.48E-01 | IVW (fixed effects) | No | No | False |
| B2 | BAD | 81 | 69 | 7.52E-01 | IVW(random effects) | Yes | No | True |
|  | Asthma2020 |  | 66 | 1.03E-01 | IVW(random effects) | Yes | No | True |
|  | ADE2015 |  | 67 | 3.98E-01 | IVW(random effects) | Yes | No | False |
|  | PA |  | 14 | 2.59E-01 | IVW(random effects) | Yes | No | False |
|  | SA |  | 14 | 3.22E-01 | IVW(random effects) | Yes | No | False |
| C2 | BAD | 57 | 44 | 8.35E-01 | IVW(random effects) | Yes | No | True |
|  | Asthma2020 |  | 44 | 8.06E-01 | IVW(random effects) | Yes | No | True |
|  | ADE2015 |  | 50 | 4.16E-01 | IVW (fixed effects) | No | No | False |
|  | PA |  | 10 | *9.46E-02* | IVW (fixed effects) | No | No | False |
|  | SA |  | 10 | 1.30E-01 | IVW (fixed effects) | No | No | False |

^a^ No. of clumped SNPs: number of independent genetic SNPs with a p-value <5×10^-8^ after clumping; ^b^ No. of SNPs in MRA: number of independent genetic SNPs used in the MR analysis for each pair of exposure and outcome; ^c^ MR p-value: p-value of the most suitable MR method; ^d^ the most suitable MR analysis used in MR analysis. Italic values indicate that the MR p-value is marginally significant.

**Table S3. Overall MR analyses with strong genetic instruments (*p*<5E-08) for causal effects of COVID-19 infection/severity or five types of ADs on peripheral blood hematological traits and immune-related cells.**

| **Exposure** | **Outcome** | **No. of clumped SNPs ^a^** | **No. of SNPs in MRA ^b^** | **MR**  ***p*-value ^c^** | **MR**  **Method ^d^** | **Heterogeneity** | **Pleiotropy** | **Directionality** |
| --- | --- | --- | --- | --- | --- | --- | --- | --- |
| ***COVID-19 infection/severity*** | | | | | | | | |
| A2 | LYMPH | 73 | 14 | **2.39E-02** | IVW(fixed effects) | No | No | FALSE |
|  | MCH |  | 14 | **4.79E-02** | IVW(fixed effects) | No | No | TRUE |
|  | CD3^+^ T |  | 16 | **1.79E-02** | IVW(fixed effects) | No | No | FALSE |
|  | CD56^+^ NK |  | 16 | **1.05E-02** | IVW(fixed effects) | No | No | FALSE |
|  | CD8^+^ T |  | 16 | **8.33E-03** | IVW(random effects) | Yes | No | FALSE |
| B2 | MCH | 81 | 13 | **4.02E-02** | IVW(fixed effects) | No | No | FALSE |
|  | CD19^+^ B |  | 16 | **1.97E-02** | IVW(fixed effects) | No | No | FALSE |
| C2 | MCH | 57 | 11 | **1.35E-02** | IVW(fixed effects) | No | No | FALSE |
| ***Allergic diseases*** | | | | | | | | |
| BAD | EOS | 144 | 32 | **1.74E-05** | IVW(fixed effects) | No | No | FALSE |
|  | CD3^+^ T |  | 39 | **2.82E-02** | IVW(fixed effects) | No | No | FALSE |
|  | CD8^+^ T |  | 39 | **3.49E-02** | IVW(fixed effects) | No | No | FALSE |
| Asthma2018 | EOS | 36 | 22 | **1.06E-06** | IVW(random effects) | Yes | No | FALSE |
|  | HB |  | 22 | **3.66E-02** | IVW(fixed effects) | No | No | TRUE |
|  | MONO |  | 22 | **5.46E-03** | IVW(fixed effects) | No | No | TRUE |
|  | CD19^+^ B |  | 31 | **6.73E-04** | IVW(fixed effects) | No | No | FALSE |
| Asthma2020 | EOS | 231 | 53 | **2.80E-07** | IVW(random effects) | Yes | No | FALSE |
|  | MONO |  | 53 | **9.53E-03** | IVW(fixed effects) | No | No | FALSE |
|  | CD19^+^ B |  | 70 | **1.11E-02** | MR Egger | Yes | Yes | FALSE |

^a^ No. of clumped SNPs: number of independent genetic SNPs with a p-value <5×10^-8^ after clumping; ^b^ No. of SNPs in MRA: number of independent genetic SNPs used in the MR analysis for each pair of exposure and outcome; ^c^ MR p-value: p-value of the most suitable MR method; ^d^ the most suitable MR analysis used in MR analysis; Bold values indicate that the MR p-value is less than 0.05.

**Table S3. Continued.**

| **Exposure** | **Outcome** | **No. of clumped SNPs ^a^** | **No. of SNPs in MRA ^b^** | **MR**  ***p*-value ^c^** | **MR**  **Method ^d^** | **Heterogeneity** | **Pleiotropy** | **Directionality** |
| --- | --- | --- | --- | --- | --- | --- | --- | --- |
| ADE2021 | EOS | 52 | 9 | **5.55E-03** | IVW(fixed effects) | No | No | FALSE |
|  | HB |  | 9 | **1.85E-02** | IVW(fixed effects) | No | No | FALSE |
|  | MCV |  | 9 | **3.98E-02** | IVW(fixed effects) | No | No | FALSE |
|  | RBC |  | 9 | **2.37E-02** | IVW(fixed effects) | No | No | FALSE |
| Shrimp allergy | LYMPH | 19 | 1 | **6.30E-06** | Wald ratio | - | - | TRUE |
|  | MONO |  | 1 | **3.33E-03** | Wald ratio | - | - | TRUE |
|  | NEUT |  | 1 | **1.85E-02** | Wald ratio | - | - | TRUE |
|  | WBC |  | 1 | **1.08E-04** | Wald ratio | - | - | TRUE |
|  | CD3^+^ T |  | 2 | **4.75E-02** | IVW(fixed effects) | No | - | TRUE |

^a^ No. of clumped SNPs: number of independent genetic SNPs with a p-value <5×10^-8^ after clumping; ^b^ No. of SNPs in MRA: number of independent genetic SNPs used in the MR analysis for each pair of exposure and outcome; ^c^ MR p-value: p-value of the most suitable MR method; ^d^ the most suitable MR analysis used in MR analysis. -: The corresponding analysis is not available; Bold values indicate that the MR p-value is less than 0.05.

**Table S4**. **MR analyses with strong genetic instruments (*p*<5E-08) for causal effects of asthma and shrimp allergy on peripheral blood ACE2 protein expression level.**

| **Exposure** | **Outcome** | **No. of clumped SNPs ^a^** | **No. of SNPs in MRA ^b^** | **MR**  ***p*-value ^c^** | **MR**  **Method ^d^** | **OR** | **LCI95** | **UCI95** |
| --- | --- | --- | --- | --- | --- | --- | --- | --- |
| Asthma2020 | ACE2-2022 | 231 | 220 | *5.74E-02* | IVW(fixed effects) | 1.02 | 1.00 | 1.05 |
|  |  |  |  | *5.22E-02* | RAPS | 1.02 | 100 | 1.05 |
| Shrimp allergy | ACE2-2017 | 19 | 3 | *8.93E-02* | IVW(fixed effects) | 0.87 | 0.75 | 1.02 |
|  |  |  |  | *9.89E-02* | RAPS | 0.87 | 0.74 | 1.03 |

^a^ No. of clumped SNPs: number of independent genetic SNPs with a p-value <5×10^-8^ after clumping; ^b^ No. of SNPs in MRA: number of independent genetic SNPs used in the MR analysis for each pair of exposure and outcome; ^c^ MR p-value: p-value of the most suitable MR method; ^d^ the most suitable MR analysis used in MR analysis. OR LCI95, lower confidence interval for OR; OR UCI95, upper confidence interval for OR; RAPS, Robust Adjusted Profile Score; Italic values indicate that the MR p-value is marginally significant.
